# Gut microbiota, probiotics and diabetes

**DOI:** 10.1186/1475-2891-13-60

**Published:** 2014-06-17

**Authors:** Aline Corado Gomes, Allain Amador Bueno, Rávila Graziany Machado de Souza, João Felipe Mota

**Affiliations:** 1 Laboratório de Investigação em Nutrição Clínica e Esportiva (Labince). Faculdade de Nutrição, Universidade Federal de Goiás, Rua 227 Qd. 68s/nº - Setor Leste Universitário, Goiânia, Goiás, Brazil; 2 Institute of Science and the Environment, University of Worcester, Henwick Grove, Worcester WR2 6AJ, UK

**Keywords:** Probiotics, Diabetes mellitus, Gut microbiota, Inflammation, Insulin resistance

## Abstract

Diabetes is a condition of multifactorial origin, involving several molecular mechanisms related to the intestinal microbiota for its development. In type 2 diabetes, receptor activation and recognition by microorganisms from the intestinal lumen may trigger inflammatory responses, inducing the phosphorylation of serine residues in insulin receptor substrate-1, reducing insulin sensitivity. In type 1 diabetes, the lowered expression of adhesion proteins within the intestinal epithelium favours a greater immune response that may result in destruction of pancreatic β cells by CD8+ T-lymphocytes, and increased expression of interleukin-17, related to autoimmunity. Research in animal models and humans has hypothesized whether the administration of probiotics may improve the prognosis of diabetes through modulation of gut microbiota. We have shown in this review that a large body of evidence suggests probiotics reduce the inflammatory response and oxidative stress, as well as increase the expression of adhesion proteins within the intestinal epithelium, reducing intestinal permeability. Such effects increase insulin sensitivity and reduce autoimmune response. However, further investigations are required to clarify whether the administration of probiotics can be efficiently used for the prevention and management of diabetes.

## Introduction

According to the World Health Organization [1], the global prevalence of diabetes is approximately 10%, reaching up to 33% of the population in some regions. Diabetes is a condition of multifactorial origin, including genetic and environmental factors, and accounts for 3.5% of the mortality cases due to non-communicable chronic diseases. Scientific evidence suggests increased inflammatory stress is related to molecular mechanisms leading to insulin resistance, and the intestinal microbiota interacts with environmental factors and susceptible genetic factors, contributing to the development of diabetes [2].

The human gastrointestinal tract contains in average 10^14^ microorganisms/ml of luminal content, and features over 5000 bacterial species. Among bacterial species, approximately 90% of them belong to the Bacteroidetes phyla, composed mainly of Gram– bacteria, and the Firmicutes phyla, composed mainly of Gram + bacteria. It has been suggested that the intestinal microbiota composition is associated with conditions such as allergies, intestinal inflammatory diseases, cancer, diabetes, cardiovascular diseases and dyslipidaemia [3,4]. It has also been suggested that altered intestinal microbiota leads to increased intestinal permeability and mucosal immune response, contributing to the development of diabetes. Increased intestinal permeability is a result of reduced expression of tight junction proteins, eventually favouring the translocation of bacterial lipopolysaccharide (LPS), which may result in metabolic endotoxemia and insulin resistance [5,6].

Modulation of intestinal microbiota by probiotics may facilitate the management of a number of clinical conditions [7]. Probiotics may be involved in the maintenance of a healthier gut microbiota, and have also been identified as effective adjuvants in insulin resistance therapies [8-10]. Hence, this systematic review appraises the current literature covering gut microbiota, probiotics and diabetes. Our aim is to clarify the currently described effects of probiotics in the prevention and management of type 1 (T1D) and type 2 (T2D) diabetes mellitus.

## Methods

A systematic literature review has been performed over the electronic databases Medline PubMed and SciELO (The Scientific Electronic Library Online). The reference list of identified articles has also been reviewed. For this search, the following descriptors were considered: "diabetes," "oxidative stress", "probiotics", "inflammation", "insulin" and "microbiota". The logical connectives "and", "or" and "and not" were systematically used to combine descriptors and terms used to trace the publications.

As a result of this appraisal conducted in December 2013, 287 publications were identified. Subsequently, the articles that met the following inclusion criteria were selected: (a) experimental studies that investigated assessment of insulin sensitivity, glucose tolerance test, intestinal permeability and or markers of oxidative stress and inflammation; (b) literature review articles; (c) articles preferably published after 2002.

After reconsideration based on the inclusion criteria, 166 articles were excluded for one of the two following reasons: the effects of probiotics were related to other pathologies other than diabetes; or, probiotics were not the main objective of the study. Therefore, 121 articles met all the inclusion criteria and were reviewed.

### Intestinal microbiota and type 2 diabetes

Muscle and adipose tissue resistance to insulin actions observed in T2D is triggered mainly by a complex combination of genetic predisposition, body composition, nutritional and environmental factors. Insulin receptor, glucose transporter and post-receptor perturbations are observed in T2D. Eventually, peripheral tissues exposed to chronic compensatory hyperinsulinemia become resistant to insulin [11]. Studies have shown the intestinal microbiota is associated with the development of metabolic diseases, as obese and diabetic subjects present perturbations in the proportions of Firmicutes, Bacteroidetes and Proteobacterias [4,12].

Mammals present sterile gastrointestinal tract at birth, and their microbiota is gradually accumulated after birth through the physical contact with the breast and the environment [13]. Infants’ intestinal microbiota is mainly formed by Bifidobacteria and Enterobacteria, and it changes progressively into a more complex pattern, observed in adults [14]. These microorganisms and their metabolites interact with the intestinal epithelial cells differently in the small and large intestines. Such microbiological and biochemical variations are attributed to the distinct anatomical features of these two organs, and also to the mucus produced by goblet cells. Mucus acts as bacterial insulator at intestinal barrier level, but does not fully impede bacterial fragments to diffuse through the intestinal barrier, binding to pattern recognition receptors (PRR). Such phenomenon contributes not only to the maintenance of the intestinal barrier but also to the innate and adaptive immune responses [15].

Diet is pivotal for regulation of the intestinal microbiota, excess of nutrients like saturated [16] and polyunsaturated fatty acids [17] or shortage of oligosaccharides [18,19] and phytochemicals [20] can modify the bacterial metabolic activity [21]. High fat diets modify the intestinal microbiota, leading to increased intestinal permeability and susceptibility to microbial antigens, which ultimately correlates with the occurrence of metabolic endotoxemia and insulin resistance [22].

The molecular mechanisms involved in high fat diets and modulation of the intestinal microbiota are not fully elucidated, but as this typical diet increases fatty acid oxidation in the liver and adipose tissue, the evidence available suggests the reactive oxygen species (ROS) generated reduce mucus production in the intestinal epithelium. Thus, the weakened intestinal barrier integrity allows the translocation of intestinal bacteria [23]. Furthermore, production of malondialdehyde as result of polyunsaturated fatty acid oxidation induces damage to the epithelial cell membranes, increasing intestinal tight junction permeability [24,25].

Diabetic individuals have lower counts of Bifidobacterium and Faecalibacterium prausnitzii, both of them Gram + [26] with anti-inflammatory properties [26,27]. Despite the perturbations already observed in the intestinal microbiota of type 2 diabetic subjects, it is still necessary to elucidate whether the variations in the microbiota, intestinal barrier and metabolic endotoxemia are causes or consequences of diabetes. Interestingly, Mehta et al. [28] showed that acute inflammation induced by intravenous administration of LPS promotes metabolic endotoxemia and systemic insulin resistance, following modulation of specific adipose inflammatory and insulin signaling pathways.

The relationship between LPS and the development of T2D has been observed in some clinical trials [29-33]. Concurrent with metabolic endotoxemia, translocation of live bacteria from the intestinal barrier into the blood appears to be related to the development of T2D [34,35].

One of the features common to metabolic diseases such as obesity and T2D is a mild chronic inflammatory state, which could possibly be – among other factors – the result of TLR activation by LPS, present in the cell wall of Gram– bacteria. The TLRs comprise a large family of cell membrane proteins present in different types of cells, recognizing microbe-associated molecular patterns (MAMPs) during the inflammatory response. TLRs play an important role in the innate immune system due to their ability to detect the presence and nature of pathogens, providing the first line of host defence. However, TLRs also stimulate adaptive immunity, once they induce the secretion of inflammatory cytokines [36]. These receptors feature leucine-rich repeat (LRR) extracellular domains, and Toll/interleukin-1 receptor (TIR) intracellular domains [37].

Toll-like receptors 4 (TLR4) are present in tissues targeted for insulin actions. Such actions may become compromised upon TLR4 stimulation, through activation of cytokine signalling cascades alongside increased concentration of reactive oxygen species (ROS) [38,39]. Reduced Bifidobacterium due to a high-fat diet intake has been associated with higher concentrations of LPS, one of the features of metabolic endotoxemia [40]. A high-fat diet intake promotes the death of Gram– bacteria, contributing to LPS production in the gut and its translocation into intestinal capillaries and the general circulation [41]. Such effect leads to higher concentrations of pro-inflammatory cytokines in various tissues via TLR4 activation [42,5,43].

Inflammation levels are pivotal for intestinal microbiota regulation and for the development of insulin resistance. The inflammatory response is activated by MAMPs such as LPS, flagellin, peptidoglycans, damage-associated molecular pattern molecules (DAMPS), high-mobility group protein (HMGB), DNA and nucleotides [36]. The recognition of DAMPS and MAMPs is mediated by specific receptors, including toll-like receptors (TLRs), C-type lectin receptors (CLRs), receptors for advanced glycation end products (RAGE), nucleotide binding oligomerization domain-like receptor (NLR) intracellular domains, and retinoic acid inducible gene type 1 (RIG-1). Activation of such receptors results in phosphorylation of c-Jun N-terminal kinases (JNKs) and IkappaB kinase complexes (IKKβ), consequently amplifying the inflammatory response [37].

As TLR4 forms a molecular complex with its soluble myeloid differentiation factor-2 (MD2) co-receptor at cell surface level, it becomes a binding site for LPS. The now formed complex TLR4-MD2-LPS triggers a cascade of inflammatory events, leading to the activation of nuclear factor kappa B (NFκB) via activation of the intracellular Toll-interleukin 1 receptor domain-containing adapter protein (TIRAP), and of the TIR domain-containing adaptor-inducing interferon-β [TRIF]-related adaptor molecule (TRAM). TIRAP and TRAM activation triggers the myeloid differentiation factor 88 (MyD88) and the TRIF pathways, respectively. The MyD88 recruits proteins of the families Interleukin-1 receptor-associated kinase (IRAK) and TNF-α receptor-associated factor 6 (TRAF6). TRAF6 is responsible for activation of the transforming growth factor β-activated kinase 1 (TAK1); activated TAK1 promotes phosphorylation of the kappa beta kinase (IKK) inhibitors α, β and γ [44].

Phosphorylated IKK complexes degrade the inhibitory kappa B (IkB), translocating the NFκB to the nucleus, subsequently inducing the expression of pro-inflammatory cytokines. Activation of inflammatory pathways induced by LPS-TLR4 increases the expression of inducible nitric oxide synthase [45], promoting the S-nitrosation/S-nitrosylation phenomenon. The generated nitric oxide reacts with cysteine residues to form adducts of S-nitrosothiols [46], inhibiting the insulin transduction signal via phosphorylation of insulin receptor 1 substrate (IRS-1) in serine, which in turn leads to insulin resistance in hepatic, muscle and adipose tissues [47,48]. Others studies have shown pro-inflammatory cytokines induce phosphorylation of IRS-1 in serine [49,50], whose activation may mediate the inhibition of insulin receptor tyrosine kinase and protein kinase B (AKT) signalling, also increasing the degradation of IRS-1 [51,52].

### Intestinal microbiota and type 1 diabetes

Type 1 diabetes results from autoimmune destruction of pancreatic β cells in genetically predisposed individuals [53]. β cell destruction involves innate and adaptive immune responses, and when around 80% of the β cells are affected, the first signs of diabetes become manifested [54]. At this point insulin therapy is mandatory.

Some epidemiological evidence – such as declined incidence of T1D in Caucasian Europeans, increased incidence in children under five in some demographic areas such as North America, Australia and North Africa, as well as T1D discordance in monozygotic twins – suggests the contribution of environmental factors for the development of this condition [55,56].

The intestinal mucosa is a major site for pathogen invasion: when undamaged, it provides the first line of defence against antigens. The intestinal wall is constituted of a layer of mucus, IgA-secreting cells, antimicrobial peptides, and a complex system of epithelial barrier formed by adhesion and tight junctions [57]. The intestinal microbiota is capable of modulating the immune response and consequently autoimmunity; the influence of intestinal bacteria in the pathogenesis of T1D has been demonstrated [58].

The mechanisms associating gut microbiota and T1D development are yet to be fully understood. Studies in humans tend to suggest this possibility; however, these results have not yet proven a direct relationship between changes in intestinal microbiota and the development of autoimmune diseases.

Increased intestinal permeability may facilitate the absorption of antigens which can injure pancreatic β cells [59]. Individuals susceptible to T1D and other autoimmune diseases present inadequately functioning intestinal barrier [60], allowing greater exposure of antigens to the immune system. However, the mechanisms resulting in this condition – leaky gut – before T1D has developed itself have not yet been fully understood. T1D patients show perturbations in the structure of tight junctions as result of decreased zonulin expression, a protein related to the regulation of intestinal permeability [61], as well as increased paracellular space between intestinal epithelial cells [6].

Antigens of dietary or pathogenic origins, facilitated by increased intestinal permeability, trigger inflammation and immune responses, which may lead to destruction of pancreatic β cells [62-64]. Bovine insulin for example, which can be found in cow’s milk, appears to sensitize intestinal T lymphocytes in susceptible children, which in turn could participate in the autoimmune destruction of pancreatic β cells [65]. Furthermore, changes in intestinal microbiota may result in altered inflammatory responses, an important event in the pathogenesis of autoimmune diseases such as T1D [66]. Children with T1D showed higher counts of Clostridium, Bacteroides and Veillonella, followed by lower counts of Bifidobacterium and Lactobacillus, than healthy children [67]. Another study reported in T1D increased counts of *Bacteroides ovatus* and decreased *Bacteroides fragilis*[68].

Intestinal pathophysiology is related to the development of T1D, once increased intestinal permeability is detectable even before the clinical onset of the disease [69]. A pioneering study by Brugman and colleagues [70] found in BioBreeding diabetes-prone (BB) rats that progressed into T1D presented reduced variety of bacteria of the phylum Bacteroidetes, as compared to control rats. Ribosomal RNA analyses of stool samples of BB and BioBreeding diabetes-resistant (BBR) rats showed increased presence of bacteroidetes and clostridium in BB rats, and increased presence of lactobacilli and bifidobacterium in BBR rats [71]. The alterations observed in BB rats may be due to limited functioning of their immune system [72]; however, this causal relationship remains under investigation.

In agreement with these results, a study [73] found that healthy children have more diverse and stable intestinal microbiota as compared to children who developed T1D. In another study, the gut microbiota composition of T1D children showed increased virulence factors, phage, prophage, motility genes and higher response to stress [74]. Corroborating these findings, it has also been found in T1D children lower counts of bacteria producing butyrate, a short chain fatty acid with anti-inflammatory actions [75]. Butyrate reduces bacterial translocation, improves the organization of tight junctions [76] and stimulates the synthesis of mucin, a glycoprotein maintaining the integrity of the intestinal epithelium [77].

MyD88 knockout mice were protected from developing T1D, and showed lower expression of TNF-α in the pancreatic lymph nodes as compared to wild-type mice [78]. The same study found that MyD88 deletion was associated with lower ratios of Firmicutes over Bacteroidetes, and increased counts of Lactobacilli, Rikenellaceae and Porphyromonadaceae. Another experimental study showed that antibiotic administration prevented insulitis and pancreatic β cell destruction in mice with virus-induced T1D through mechanisms involving reduction of the innate immune response in pancreatic lymph nodes and Peyer's patches [79]. These results support the hypothesis that the innate immune system is related to the development of T1D.

In contrast, intestinal microbiota destruction of MyD88 knockout rats by broad-spectrum antibiotics was associated with increased incidence of T1D, as compared to germ-free mice [78]. This result suggests that specific components of the intestinal microbiota may prevent the activation of autoimmune T cells independently of the presence of MyD88 in ways not yet fully understood.

Studies in T1D animal models have elucidated several pathogenic pathways that may lead to immune-mediated destruction of β cells. CD8+ cells, for example, can destroy β cells via perforin expression. The presence of proinfammatory cytokines induces damage to β cells, and molecules of the TNF family induce apoptosis [80].

Studies in humans have also shown involvement of the immune system in the destruction of pancreatic β cells. Interferons produced in inflammatory and infectious responses accelerate the destruction of pancreatic β cells by inducing the expression of MHC class I [81]. Higher expression of MHC class I epitopes [82] and CD8+ T cells [83] have been observed in the pancreas of T1D individuals. CD4+ and CD8+ T cells are related to the pathogenesis of T1D, once CD4+ may invade pancreatic islets, and CD8+ may initiate β cell destruction [84].

Studies focusing on specific bacterial lineages have revealed that *Bacteroides fragilis*, a member of the Bacteroidetes phyla [85], present the ability to reduce intestinal inflammation, whilst segmented filamentous bacteria are able to activate IL-17-producing CD4+ T helper cells (TH17), which stimulate autoimmune responses and the production of inflammatory cytokines [86-88]. Interestingly, TH17 induction is dependent upon the individual’s genetic background [86].

It has been proposed the maintenance of normal microbiota – the 'old friends hypothesis’ – is promoted by lower modulatory levels of regulatory T cells secreting IL-10 and transforming growth factor beta (TGFβ), which decrease inflammation [89]. Geuking et al. [86] proposes this mutualistic response to be related to a variety of regulatory T cell subsets in a complex real-life gut flora, inclusive of symbiotic, commensal with the potential to become pathogenic, and pathogenic microorganisms. In that complex scenario, further research is needed to specify which Bacteroidetes species reduce intestinal inflammation and promote regulatory T cells induction. Taken together however, these responses support the maintenance of self-tolerance, and suggest an important role of probiotics in maintaining a healthier intestinal microbiota [64].

### Evidences from experimental and clinical studies

Probiotics are a class of live microorganisms which, when ingested in appropriate amounts, may confer health benefits to their host [90]. Consumption of probiotics may be associated with immune system stimulation, decreased cholesterol blood levels, protection against respiratory and intestinal diseases, reduction of inflammatory responses and antitumorigenic effects. These alleged health claims apparently stem from the ability of probiotics to secrete antimicrobial substances, competing with other pathogens, strengthening the intestinal barrier and modulating the immune system [91]. Bifidobacteria and lactobacilli are the most commonly used strains in functional foods and dietary supplements [92]. A summarized list of studies evaluating the effects of probiotic administration in experimental models and clinical investigations in diabetes mellitus is presented in Tables 1 and 2.

**Table 1:** Effects of probiotic administration on diabetes mellitus – Experimental studies

**References**	**Probiotics**	**Type of cell/animal model**	**Quantity**	**Study period**	**Results**
[34]	*Bifidobacterium animalis subsp. lactis* 420	C57BL/6, ob/ob, CD14-/-, ob/obxCD14-/-, Myd88-/-, Nod1-/-or Nod2-/-mice fed a high fat diet	10^9^ CFU/day	6 weeks	↓ TNF-α, IL-1β, PAI-1 and IL-6
					↑Insulin sensitivity
[94]	*L. acidophilus* (PZ 1041)*, L. gasseri* (PZ 1160), *L. fermentum* (PZ 1162), and *L. rhamnosus* (PZ 1121)	T84 cell	-	-	*L. acidophilus*, *L. fermentum*, and *L. gasseri*: ↑ two genes encoding adherence junction proteins β-catenin and E-cadherin
					↑ Transepithelial electrical resistance
					↓ PKCδ
[96]	*Lactobacillus johnsonii* N6.2 subsp. infantis ATCC	Caco-2 cell/BB rats	10^10^ to 10^11^ CFU/L in cell culture and 10^8^ CFU/day in rats	-	↑ Paneth cell
[99]	*Lactobacillus johnsonii* N6.2 and *Lactobacillus reuteri* TD1	BB rats	10^8^ CFU/day	141 days	*Lactobacillus johnsonii N6.2:* ↓ incidence of T1D; ↑ goblet cells and claudin-1; ↓ hexanoyl-lysine (oxidative stress biomarker)
[100]	*Lactobacillus johnsonii* N6.2 and *Lactobacillus reuteri* TD1	BB rats, NOD mice, and C57BL/6 mice	1 × 10^8^ CFU/day	140 days	Positive TH17 phenotype modulation
[102]	*L. acidophilus, L. casei,* and *L. lactis*	Male Wistar rats fed a high fructose diet	diet supplemented with 15% of dahi *ad libitum*	8 weeks	↓ Blood glucose, HbA1c, glucose intolerance, plasma insulin, liver glycogen, plasma total cholesterol, triacylglycerol, low-density lipoprotein cholesterol, very low-density lipoprotein cholesterol, and blood free fatty acids
[103]	*L. plantarum* DSM 15313	Female C57BL/6 J mice fed a high fat diet	25 × 10^8^ CFU/day	20 weeks	↓ Blood glucose
[104]	*VSL#3 (L. acidophilus* MB 443*, L. delbrueckii subsp. bulgaricus* MB 453*, L. casei* MB 451*, L. plantarum* MB 452*, B. longum* Y10*, B. infantis Y1, B. breve* Y8*,* and *S. salivarius subsp. thermophilus* MB 455)	ApoE-/-C57BL6 male mice	25 × 10^8^ CFU/day	12 weeks	↓ Insulin
					↑ Glucose tolerance
					↑ Insulin signaling
					↓TNF-α and RANTES
					↑ IL-10
[105]	*VSL#3 (L. acidophilus* MB 443*, L. delbrueckii subsp. bulgaricus* MB 453*, L. casei* MB 451*, L. plantarum* MB 452*, B. longum* Y10*, B. infantis Y1, B. breve* Y8*,* and *S. salivarius subsp. thermophilus* MB 455)	NOD mice	1.5 × 10^9^ CFU/day	12 weeks	↓ Hepatic NKT cell depletion
					↓ IKKβ activity
					↓ NF-κB binding activity
					↑Insulin signaling
[106]	*VSL#3 (L. acidophilus* MB 443*, L. delbrueckii subsp. bulgaricus* MB 453*, L. casei* MB 451*, L. plantarum* MB 452*, B. longum* Y10*, B. infantis Y1, B. breve* Y8*,* and *S. salivarius subsp. thermophilus* MB 455)	Female NOD mice	9 mg/week	70 weeks	↓ Incidence of auto-immune diabetes
					↓ Insulitis and decreased rate of β-cell destruction
					↑ IL-10
[109]	*Lactococcus lactis ssp. diacetylactis* NCDC 60*, L. acidophilus* NCDC 14, and *L. casei* NCDC 19	Male Wistar diabetic rats	15 g/day (8,83 CFU/g lactobacilli and 7,89 log CFU/g lactococci)	15 weeks	↑ Gastric emptying
					Dahi probiotic feeding did not change blood glucose levels
					↓ Thiobarbituric acid-reactive species in intestinal tissues
					↓ HbA1c
[111]	*L. reuteri* GMNL-263	Male Sprague–Dawley diabetic rats	1 × 10^9^ CFU/day	4 weeks	↓ HbA1c and blood glucose
					↓ JAK2 and STAT1 phosphorylation
					↓ PAI-1
[112]	*Bifidobacterium adolescentis*	Male Wistar rats fed a high fat diet	-	12 weeks	↓ Body weight
					↑Insulin sensitivity
[114]	*Lactobacillus rhamnosus GG*	HT-29 cells	10^7^-10^9^ CFU/mL	-	↓ NF-kB nuclear translocation
					↓ LPS-induced IκBα degradation

HbA1c: Glycated hemoglobin; NF-kB: nuclear factor kappa B; LPS: Lipopolysaccharides; IκBα: inhibitory kappa B alpha; TNF-α: tumor necrosis factor alpha; IL-1β: interleukin-1 beta; PAI-1: plasminogen activator inhibitor-1; IL-6: interleukin-6; JAK2: Janus kinase 2; STAT1: signal transducer and activator of transcription 1; IL-10: interleukin-10; IKKβ: inhibitors of kappa beta kinase beta; NKT: natural killer T cells; RANTES: regulated upon activation, normal T-cell expressed and secreted; Th17: T helper 17; T1D: type 1 diabetes.

**Table 2:** Effects of probiotic administration on diabetes mellitus – clinical studies

**References**	**Probiotic**	**Study design/subjects**	**Sample Size**	**Quantity**	**Study period**	**Results**
[8]	*Lactobacillus acidophilus* and *Bifidobacterium bifidum*	Double-blinded, placebo-controlled, randomized study, T2D females aged 50–65 years	Placebo group: n = 10; Probiotic group: n = 10	2 daily doses of 100 mL symbiotic shake containing 4 × 10^8^ CFU/100 mL *Lactobacillus acidophillus*, 4 × 10^8^ CFU/100 mL *Bifidobacterium bifidum*	45 days	↓ Glycemia
[9]	*Lactobacillus acidophilus* La5 and *Bifidobacterium lactis* Bb12	Double-blinded, randomized controlled clinical trial, T2D patients aged 30–60 years	Placebo group: n = 32; Probiotic group: n = 32	300 g/day of probiotic and conventional yogurt day 1: 7,23 × 10^6^ of *L. acidophilus* La5 and 6.04 × 10^6^ cfu/g of *B. lactis* Bb12	6 weeks	↓ Fasting blood glucose and HbA1c
						↑ Erythrocyte SOD and GPx
						↑ Total antioxidant capacity
[10]	*L. acidophilus* NCFM	Double-blinded, placebo-controlled, randomized study, T2D males	Placebo group: n = 24; Probiotic group: n = 24	-	4 weeks	Preserved insulin sensitivity
						No effect on systemic inflammatory response
[117]	*Lactobacillus rhamnosus* GG (ATCC 53 103) and *Bifidobacterium lactis* Bb12	Prospective, randomized study, mother–baby pairs	Dietetic Intervention + probiotics: n = 85; Dietetic Intervention + placebo: n = 86; Control + placebo: n = 85	Lactobacillus rhamnosus GG: 10^10^ CFU/day; Bifidobacterium lactis Bb12: 10^10^ CFU/day	33 months	↓ Risk of GDM
[118]	*Lactobacillus rhamnosus* GG, ATCC 53 103 and *Bifidobacterium lactis* Bb12	Randomized, prospective, parallel-group, combined dietary counselling, pregnant women	Diet + probiotics: n = 85; Diet + placebo: n = 86; Control + placebo: n = 85	Lactobacillus rhamnosus: 10^10^ CFU/day; Bifidobacterium lactis Bb12: 10^10^ CFU/day	18 months	↓ Blood glucose
						↓ Insulin
						↓ Insulin sensitivity
[119]	*L. plantarum* WCFS1	Double-blinded, randomized crossover study, healthy subjects	n = 14	10^12^ CFU	6 hours	↓ Degradation of transepithelial electrical resistance
						↑ ZO-1 in tight junctions

GDM: gestational diabetes mellitus; GPx: Glutathione peroxidase; HbA1c: Glycated hemoglobin; SOD: Superoxide dismutase; T2D: type II diabetes mellitus; ZO-1: zonula occludens-1.

It has been shown that *Lactobacillus acidophilus, L fermentum, L gasseri and L rhamnosus* modulate the expression of genes encoding junction and adhesion proteins E-cadherin and β-catenin, and reduce the expression of protein kinase C-δ (PKC-δ) [93]. PKC-δ activation results in dispersion of adherence junctions, increasing intestinal permeability [94]. On the other hand, β-catenin phosphorylation induced by probiotics may strengthen the complex E-cadherin/β-catenin, supporting the maintenance of adhesion junction from the binding site of E-cadherin to the cytoskeleton [95].

The administration of 10^8^ colony-forming units (CFU) of *Lactobacillus johnsonii* N6.2 per day to rats increased the number of Paneth cells [96]. Paneth cells are constituents of the intestinal barrier which produce antimicrobial proteins, contributing to reduce intestinal permeability [97]. Experimental studies have shown the administration of *Lactobacillus johnsonii* to diabetes-prone rats may reduce the incidence of diabetes by increasing the gene expression of claudin-1 and decreasing oxidative stress [98], and it may also modulate the TH17 response, impairing the development of T1D in diabetes-prone mice [99]. Several microbial species possess the ability to modulate the TH17 phenotype, particularly Gram + bacteria [100]. Even though TH17 induces pancreatic inflammation, progression to T1D will occur only after this cell differentiates into TH1; so apparently the formation of differentiated TH17 phenotypes appears to inhibit the manifestation of the diabetogenic phenotype [101].

The consumption of dahi, a traditional Indian fermented milk, containing *L. acidophilus, L. casei* and *L. lactis* has been shown to reduce the glycemic curve and HbA1c [102]. *L. plantarum* DSM15313 is also suggested to reduce glycaemia, improve glucose tolerance and reduce insulin resistance [103]. VSL#3, a commercially available mixture of probiotics containing 3 × 10^11^ CFU/g of *Bifidobacterium longum*, *B. infantis* and *B. breve,* has been shown to improve insulin signalling and reduce inflammation in the adipose tissue of ApoE-/- rats [104]. VSL#3 has been also shown to reduce the depletion of hepatic natural killer cells and minimize the activation of NFkB in wild-type male C57BL6 mice fed a high fat diet [105]. A study investigating the effects of VSL#3 on the occurrence of diabetes in non-obese diabetic mice showed this probiotic mixture has impaired the development of T1D via three major pathways: 1) supressing both inflammation and pancreatic β cell death, 2) increasing the production of IL-10 from Peyer’s patches, a component of the gut-associated lymphoid tissue, and 3) increasing the IL-10 expression in the pancreas [106]. IL-10 is an anti-inflammatory cytokine which inhibits antigen presentation and proinflammatory cytokine production [107], whereas depletion of hepatic natural killer cells is linked to the development of hepatic insulin resistance [108].

Consumption of dahi enriched with *Lactobacillus acidophilus* NCDC14 and *Lactobacillus casei* NCDC19 has apparently reduced lipid peroxidation, HbA1c and ameliorated intestinal transit in diabetic rats; however, without concomitant blood glucose reduction [109]. Similar study has shown preserved enzymatic activity of the antioxidant enzymes glutathione peroxidase, superoxide dismutase and catalase [110]. The administration of *Lactobacillus reuteri* GMNL-263 has been shown to reduce glycaemia and HbA1c levels, and to prevent renal fibrosis [111]. It has also been suggested that *Bifidobacterium adolescentis* improves insulin sensitivity [112] via increased production of glucagon-like peptide 1 (GLP-1) [2]. GLP-1 enhances glucose tolerance via complex mechanisms involving modulation of insulin secretion, pancreatic cell mass and food intake [113].

Administration of 10^9^ CFU of *Bifidobacterium animalis ssp. lactis* 420 to high fat diet-fed diabetic rats appears to reduce the inflammatory cytokines TNF-α, IL-1β, plasminogen activator inhibitor-1 (PAI-1) and IL-6 in mesenteric adipose tissue, as well as to improved insulin sensitivity [34]. In the same way, *Lactobacillus rhamnosus* GG appears to reduce translocation of NFkB to the nucleus, degradation of Ikβ, and activation of the TLR4 by LPS [114].

Intestinal microbiota modulation by probiotics appears to offer beneficial outcomes to insulin-resistant individuals via mechanisms both related and unrelated to inflammation [115]. However, the effectiveness of clinical trials employing probiotics may be specific to the strain used and cannot be extrapolated to other strains or species [116].

A study has shown daily consumption of 200 ml of a shake containing 4 × 10^8^ CFU/100 ml of *Lactobacillus acidophilus*, 4 × 10^8^ CFU/100 ml of *Bifidobacterium bifidum* and 1 g/100 ml of fructooligosaccharides, resulted in blood glucose reduction in T2D individuals [8]. It has also been shown that T2D individuals, after consuming a yogurt containing 7,23 × 10^6^ CFU of *L. acidophilus* La5 and 6,04 × 10^6^ CFU of *B. lactis* Bb12 for 6 weeks, presented reduced fasting glucose and reduced HbA1c levels, followed by higher activity of superoxide dismutase and glutathione peroxidase, as compared to a control group [9].

The administration of capsules containing 10^10^ CFU of *L. acidophilus* NCFM for 4 weeks has preserved insulin sensitivity in a sample population of non-diabetic and diabetic individuals [10]. Pregnant women receiving intensive nutritional counselling and a food supplement containing 10^10^ CFU of *L. rhamnosus* GG and 10^10^ CFU of *B. lactis* Bb12 presented improved glucose tolerance and reduced HbA1c levels in relation to a control group receiving only a control, healthy diet [117]. A similar study from the same research group observed that nutritional counselling combined with probiotic supplementation reduced blood glucose during pregnancy and up to 12 months after delivery, reduced insulin concentrations and improved insulin sensitivity more effectively than nutritional counselling alone [118].

A study has shown the consumption of 10^12^ CFU of *Lactobacillus plantarum* WCFS1 improved the localization of the scaffolding protein zonula occludens (ZO)-1 in areas surrounding the tight junctions [119]. Accumulation of these proteins is associated with increased protection at intestinal barrier level [120], and the organization of these occlusion proteins may occur via activation of TLR2 receptors on the apical surface of the intestinal epithelium [119].

**Figure 1:**
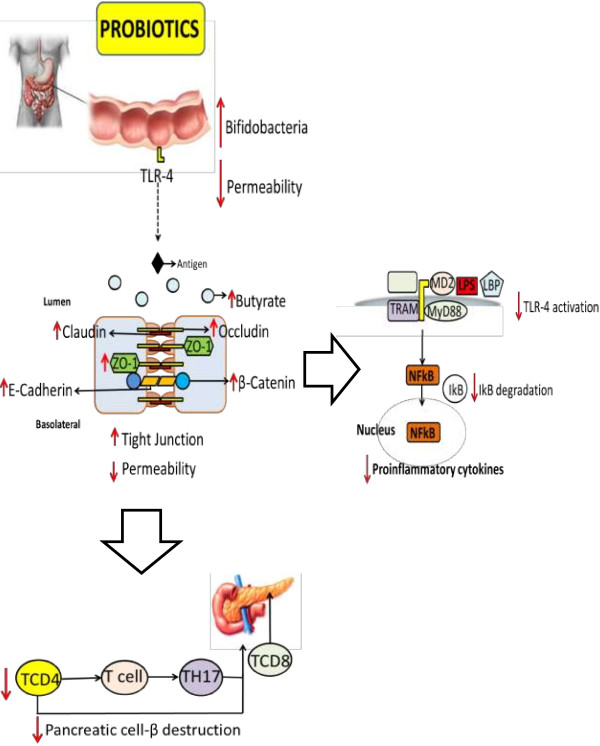
**Schematic representation of probiotic actions in type 1 and type 2 diabetes.** Probiotic consumption increases the number of bifidobacteria, and increased expression of adhesion proteins reduces intestinal permeability, impairing the activation of TLR4 by LPS. As result, NFkB activation pathways are blocked. The induction of TH17 cells is also inhibited, preventing pancreatic infiltration of CD8+ T cells.

Molecular mechanisms involving the anti-diabetic effects of probiotics are not fully elucidated, but may be related to reduction of oxidative stress, immunomodulation, attenuation of inflammation and modification of the intestinal microbiota (Figure 1) [9]. Furthermore, probiotics have been shown to improve the absorption of antioxidants and reduce post-prandial lipid concentrations, actions directly related to oxidative stress [121].

## Conclusions

The intestinal microbiota presents a vast set of antigens which may participate in the modulation of immunological diseases. An intestinal barrier presenting full integrity ensures specific interactions between the luminal antigens and the host. Functional disarrangements may contribute to the autoimmune destruction of pancreatic β cells, which leads to T1D, and increased expression of inflammatory cytokines may lead to insulin resistance and T2D.

The evidence available from experimental studies and clinical trials supports our suggestion that the modulation of the intestinal microbiota by probiotics may be effective towards prevention and management of T1D and T2D. The findings discussed here provide an insight into the investigation of further hypotheses aiming to elucidate molecular mechanisms involved in the modulation of intestinal microbiota by probiotic administration, their roles on the development of T1D and T2D and potential effectiveness for clinical practice.

## Abbreviations

AGE: Advanced glycation end products; AKT: Protein kinase B; AP-1: Activating protein 1; BB: BioBreeding diabetes-prone; BBR: BioBreeding diabetes-resistant; CFU: Colony-forming units; CLRs: C-type lectin receptors; CREB: CRE binding protein; DAMPS: Damage-associated molecular pattern molecules; FFA: Free fatty acid; GLP-1: Glucagon-like peptide 1; GSK-3: Glycogen synthase kinase 3; HbA1c: Glycosylated haemoglobin; HMGB: High-mobility group protein; IkB: Inhibitory kappa B; IKK: Inhibitors of kappa beta kinase; IKKβ: Inhibitors of kappa beta kinase beta; IRAK: Interleukin-1 receptor-associated kinase; IRF3: Interferon-regulatory factor 3; IRS-1: Insulin receptor 1 substrate; JNKs: c-Jun N-terminal kinases; LPS: Lipopolysacharide; LRR: Leucine-rich repeat; MAPK-p38: Mitogen-activated protein kinase p38; MD2: Myeloid differentiation factor 2; MyD88: Myeloid differentiation factor 88; NFκB: Nuclear factor kappa B; NOD: Nucleotide-binding oligomerization domain receptor; NLR: Nucleotide binding oligomerization domain-like receptor (NLR) PAI-1, plasminogen activator inhibitor-1; PKC: Protein kinase C; PRR: Pattern recognition receptors; RAGE: Receptors for advanced glycation end products; RIG-1: Intracellular domains, and retinoic acid inducible gene type 1; ROS: Reactive oxygen species; SciELO: The Scientific Electronic Library Online SOCS3, suppressor of cytokine signalling 3; T1D: Type 1 diabetes mellitus; T2D: Type 2 diabetes mellitus; TAK1: Transforming growth factor β-activated kinase 1; TIR: Toll/interleukin-1 receptor; TIRAP: Intracellular Toll-interleukin 1 receptor domain-containing adapter protein; TLRs: Toll-like receptors; TLR4: Toll-like receptor 4; TNF-α: Tumor necrosis factor alpha; TRAF6: TNF-α receptor-associated factor 6; TRAM: TRIF-related adaptor molecule; TRIF: TIR-domain-containing adaptor-inducing interferon-β; ZO: Zonula occludens.

## Competing interests

The authors declare that they have no competing interests.

## Authors’ contributions

ACG drafted the manuscript and performed the design of the study. AAB, RGMS and JFM drafted and revised the manuscript. All authors have read and approved the final version of this manuscript.

## Authors’ information

ACG: Graduated in Nutrition, Federal University of Goiás (2011), MSc in Nutrition and Health, Federal University of Goiás.

AAB: Graduated in Biological Sciences Medical Modality, Federal University of São Paulo (2000), MPhil in Nutrition Sciences, Federal University of São Paulo (2003), PhD in Nutrition Sciences, Federal University of São Paulo, Post-doctoral researcher in Nutritional Biochemistry, Institute of Brain Chemistry and Human Nutrition, London, currently Senior Lecturer at the Institute of Science and the Environment University of Worcester.

RGMS: Graduated in Nutrition, Federal University of Goiás (2008), MSc in Nutrition and Health, Federal University of Goiás.

JFM: Graduated in Nutrition, Pontifícia Universidade Católica de Campinas (2002), MSc in Pathology, São Paulo State University (2007), PhD in Nutrition, Federal University of São Paulo (2011), member of the Department of Nutrition and Metabolism of the Brazilian Diabetes Society, currently Senior Lecturer at Federal University of Goiás.
